# The Principles and Practice of Hydrotherapy

**Published:** 1904-07

**Authors:** 


THE PRINCIPLES AND PRACTICE OF HYDROTHERAPY. A Guide to the Application of Water in Disease. By SIMON BARUCH, M.D., Professor of Hydrotherapeutics in the New York Post-Graduate Medical School and Hospital. Octavo, pp. 506. Illustrated. New York: William Wood & Company. 1903. (Price, $4.00.)

In the present revised edition of this work the author's aim is to help physicians to attain a real knowledge of scientific hydrotherapy. He treats the subject in a systematic manner, points out limitations and probabilities, and records actual achievements. He tells us many things about the scientific employment of water in the treatment of disease and presents in a convenient form, and unconventional style of language a certain number of elementary facts, ideas, and suggestions which the ordinary practitioner, laying no claim to hydrotherapeutic attainments generally, is usually glad to know.

The book is divided into two parts. The first is mainly theoretical in that it deals with the principles which underlie the application of water, and includes the most recent views upon the physiological effects of water, functions of the skin, rationale of the action of water in health, and its reaction.

The second part contains technical and clinical chapters. Details of the most useful methods are given as a guidance to the skilful, beneficial and effective application of water in disease. It is plain that Dr. Baruch's success in the treatment of the various diseases mentioned depends upon experience and judiciousness of method.

Whoever has the opportunity of extensive observation of this subject may not only find in it the justification of publishing his views, but an obligation to present them to the profession for the furtherance of both scientific and humanitarian progress. There are numerous good illustrations in the book.

